# Vaccinia and Smallpox

**Published:** 1855-07

**Authors:** 


*Vaccinia and Smallpox.*—The following case is so similar to one recently published in the London Lancet, and is itself of such unusual character, that you may think it worth insertion in the Medical and Surgical Journal.

A nursing infant of Mrs. Q———, some 8 or 9 months old, was vaccinated by myself, after it had been exposed to the contagion of a mild case of varioloid several days. The operation was successful, two perfect vesicles being the result; and on the seventh day took virus from the arm, and with it vaccinated two other children. On the day immediately succeeding, viz., the eighth, a papular eruption appeared upon the infant, which as it developed itself assumed all the characters of unmistakeable smallpox. The eruption was very full, as full as possible without being confluent, and the disease went on to a fatal termination. The vaccine vesicles, perfectly normal in their character at the time that virus was taken from them, from that day ceased to follow the usual course. They became large, irregular and flattened pustules, accompanying the variola in its development. The children vaccinated with matter from this patient had *genuine vaccine vesicles*, without any unusual disturbance or breaking out on the skin.

The following points are particularly noticeable in the above case:

1. The infant must have had latent variola at the time of vaccination.

2. The vaccinia was able to establish itself locally to such a degree as to extinguish at the points vaccinated the latent disorder up to the eighth day.

3. After this period the variola overwhelmed and engulfed, as it were, the vaccinia, and was able to expend its full force upon the system of the patient. Respectfully yours, S. L. Abbot.

Boston, May, 1855.—*Boston Med. and Surg. Journal.*

